# Stroke awareness decreases prehospital delay after acute ischemic stroke in korea

**DOI:** 10.1186/1471-2377-11-2

**Published:** 2011-01-06

**Authors:** Young Seo Kim, Sang-Soon Park, Hee-Joon Bae, A-Hyun Cho, Yong-Jin Cho, Moon-Ku Han, Ji Hoe Heo, Kyusik Kang, Dong-Eog Kim, Hahn Young Kim, Gyeong-Moon Kim, Sun Uk Kwon, Hyung-Min Kwon, Byung-Chul Lee, Kyung Bok Lee, Seung-Hoon Lee, Su-Ho Lee, Yong-Seok Lee, Hyo Suk Nam, Mi-Sun Oh, Jong-Moo Park, Joung-Ho Rha, Kyung-Ho Yu, Byung-Woo Yoon

**Affiliations:** 1 Department of Neurology, Hanyang University Hospital, Seoul, Republic of Korea; 2 Department of Neurology, Seoul National University Hospital, Seoul, Republic of Korea; 3 Department of Neurology, Seoul National University Bundang hospital, Seongnam, Republic of Korea; 4 Department of Neurology, St. Mary's hospital, Seoul Korea; 5 Department of Neurology, Inje University Ilsan Paik Hospital, Goyang, Republic of Korea; 6 Department of Neurology, Yonsei University Severance Hospital, Seoul, Republic of Korea; 7 Department of Neurology, Eulji General Hospital, Seoul, Republic of Korea; 8 Department of Neurology, Dongguk University International Hospital, Koyang, Republic of Korea; 9 Department of Neurology, Konkuk University Hospital, Seoul, Republic of Korea; 10 Department of Neurology, Samsung medical center, Seoul, Republic of Korea; 11 Department of Neurology, Asan medical center, University of Ulsan, Seoul, Republic of Korea; 12 Department of Neurology, Seoul National University Boramae hospital, Seoul, Republic of Korea; 13 Department of Neurology, Hallym University Sacred Hospital, Anyang, Republic of Korea; 14 Department of Neurology, Soonchunhyang University Hospital, Seoul, Republic of Korea; 15 Department of Neurology, Seoul-Daehyo Hospital, Seoul, Republic of Korea; 16 Department of Neurology, Inha University Hospital, Incheon, Republic of Korea

## Abstract

**Background:**

Delayed arrival at hospital is one of the major obstacles in enhancing the rate of thrombolysis therapy in patients with acute ischemic stroke. Our study aimed to investigate factors associated with prehospital delay after acute ischemic stroke in Korea.

**Methods:**

A prospective, multicenter study was conducted at 14 tertiary hospitals in Korea from March 2009 to July 2009. We interviewed 500 consecutive patients with acute ischemic stroke who arrived within 48 hours. Univariate and multivariate analyses were performed to evaluate factors influencing prehospital delay.

**Results:**

Among the 500 patients (median 67 years, 62% men), the median time interval from symptom onset to arrival was 474 minutes (interquartile range, 170-1313). Early arrival within 3 hours of symptom onset was significantly associated with the following factors: high National Institutes of Health Stroke Scale (NIHSS) score, previous stroke, atrial fibrillation, use of ambulance, knowledge about thrombolysis and awareness of the patient/bystander that the initial symptom was a stroke. Multivariable logistic regression analysis indicated that awareness of the patient/bystander that the initial symptom was a stroke (OR 4.438, 95% CI 2.669-7.381), knowledge about thrombolysis (OR 2.002, 95% CI 1.104-3.633) and use of ambulance (OR 1.961, 95% CI 1.176-3.270) were significantly associated with early arrival.

**Conclusions:**

In Korea, stroke awareness not only on the part of patients, but also of bystanders, had a great impact on early arrival at hospital. To increase the rate of thrombolysis therapy and the incidence of favorable outcomes, extensive general public education including how to recognize stroke symptoms would be important.

## Background

Despite current evidences supporting the time extension of intravenous recombinant tissue plasminogen activator (rt-PA) therapy in acute ischemic stroke patients, it is well known that early administration of thrombolytics is beneficial for patient outcome [1]. Since intravenous rt-PA is the only approved treatment for acute ischemic stroke, shortening the time between symptom onset and hospital arrival is important. Although, many interventions to reduce prehospital delay have been conducted, 3-8.5% of all stroke patients receive thrombolytic therapy in the US [2] and only 2.1% in Korea [3].

There have been numerous studies of the factors associated with prehospital delay and some factors such as contacting the primary physician, or not using Emergency Medical Services (EMS), were in almost all cases found to be associated with delayed arrival time [4,5]. However, findings concerning the impact of demographics and clinical factors, as well as of knowledge about stroke, were somewhat inconsistent, perhaps due to differences in location, time of investigation and medical environment. In one study, perceptual, social and behavioral factors, rather than knowledge, were suggested to be important for decreasing arrival delay [6], and the role of the bystander in delivery of acute stroke was highlighted. South Korea has a unique culture in which family relationships tend to be close and are held in high regard, and where relatives often live together. In addition, knowledge about acute stroke treatment is not widespread, especially among the elderly and people with little education [7]. Therefore, the results of large studies in other countries may not be applicable in Korea, and it is likely that rapid reactions on the part of bystander after the onset of stroke symptoms may be extremely important.

The aims of this study were to identify factors that influence hospital arrival time after an acute ischemic stroke, and to investigate whether awareness of stroke symptoms not only on the part of patient but also of the bystander, has a significant impact on reducing prehospital delay.

## Methods

This study was designed as a prospective, multicenter, consecutive characterization, which was conducted at 14 tertiary hospitals in Seoul, Korea, and the surrounding metropolitan area. This area has a population of approximately 24 million, and study hospitals were selected to represent different geographic locations that were primarily responsible for all stroke patients in these locations. The inclusion criteria were patients with neurologic symptoms who were hospitalized at the study hospitals and diagnosed with non-traumatic ischemic stroke by diffusion magnetic resonance imaging (MRI). Patients who visited the hospital within 48 hours of symptom onset, and were above 19 were recruited. Exclusion criteria were diagnoses of intracerebral hemorrhage, subarachnoid hemorrhage, in-hospital stroke, or lesion-negative transient ischemic attack (TIA) and patients who had been treated by thrombolysis before visiting the study hospitals. There were no public campaigns or educational efforts before or during the study. The study was approved by the institutional review board (IRB) of each hospital.

The study was performed from March to July 2009, and we decided to stop recruiting when the total number of patients reached 500. At each hospital, all of the patients who arrived at the Emergency Department (ED) and were admitted to the stroke unit were reviewed to confirm eligibility. When patients were deemed eligible, research nurses who had been formally trained in standardized definitions and data collection techniques asked them to participate in the study. The patients were interviewed within 48 hours of admission after providing informed consent. Patients who did not consent to an interview were excluded. If a patient was not able to communicate, a bystander who had witnessed the patient's symptom onset and could describe the exact arrival process was interviewed. However, patients who died soon after admission or were hospitalized in the intensive care unit were excluded because they were not available for interview. Demographic characteristics and circumstances from the onset of symptoms to arrival at the study hospital were recorded, and clinical information about the patients, such as the National Institute of Health Stroke Scale (NIHSS), was obtained from medical records. Patients were registered consecutively using the web-based database electronic Case Reporting Form (e-CRF), which was available at all the study hospitals.

Prehospital delay was defined as the time from symptom onset to arrival at the ED of the study hospital. If the symptoms occurred during sleep, the time of awakening was recorded as the time of onset because it represented the time when medical help could be sought. For patients who were referred by other hospitals or primary care physicians, arrival times and transportation methods to the referral hospital were also recorded.

Patient baseline characteristics were represented by median (interquartile range), number and proportion values. Because the distribution of the prehospital delay times was positively skewed, time differences according to explanatory variables were represented by median and 25^th ^and 75^th ^percentile values, and the Mann-Whitney *U *test was used for univariate analysis. Patients were then divided into an early arrival group (≤ 3 hours) and a late arrival group (> 3 hours), and the explanatory variables were compared by Pearson's χ^2 ^test and the Mann-Whitney *U *test. Because current evidence supports a change in the thrombolysis indication time from 3 to 4 hours 30 minutes, we thought it reasonable to dichotomize by 3-hour periods. Finally, multivariable logistic regression analysis was used to analyze the factors independently associated with prehospital delay. Explanatory variables, which were identified by univariate analysis at P < 0.2, were selected and entered into the models. All significance tests were 2-tailed, and differences were considered to be statistically significant at P < 0.05. Data were analyzed with SPSS version 12.0 for Windows (SPSS Inc).

## Results

**Figure 1:**
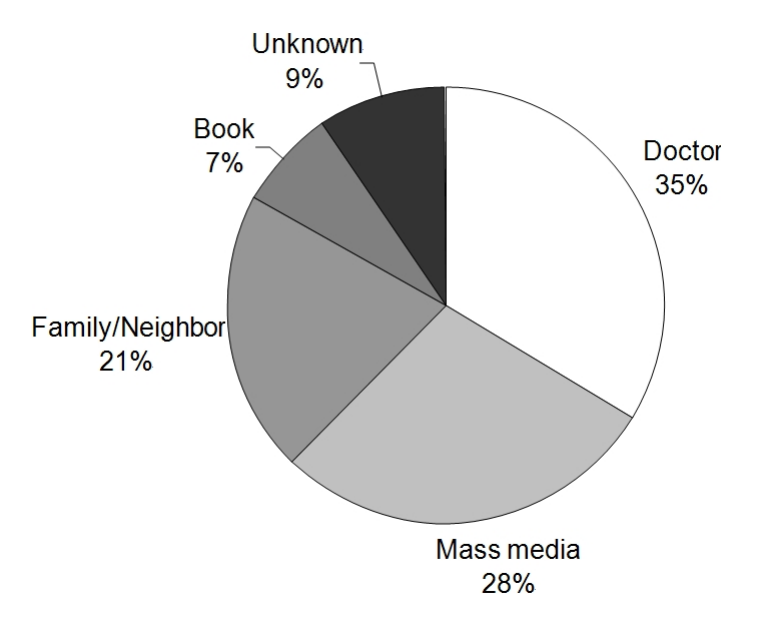
**Knowledge sources of thrombolysis**.

Of the 500 patients who were registered in the study, 62% were male, and the median age was 67. The median prehospital delay time was 474 minutes (interquartile range, 170 to 1313 minutes). One hundred and two patients (21%) arrived within 2 hours, 131 (26%) within 3 hours and 215 (43%) within 6 hours. Thirty patients (6%) underwent thrombolysis. Table 1 shows the baseline characteristics of the study population and the frequencies of the factors considered as explanatory variables for delayed arrival. One hundred and sixty-nine patients (34%) were referred from other hospitals and 182 (36%) used ambulances. The majority of cases occurred at home (71%), and 145 (29%) patients were alone at the time of symptom onset. Only 92 (18%) patients had knowledge of thrombolysis. The media and family/neighbors made up the largest sources of information (Figure 1). Of the 92 patients who knew about thrombolysis, 80 (87%) replied that it should be performed as soon as possible or within 3 hours. Stroke awareness on the part of patient or bystander was present in only 179 cases (36%), and 140 (28%) patients responded that they had never heard of strokes. When educational level was analyzed according to knowledge of thrombolysis and stroke awareness, highly educated patients (≥ 12 years) had more and correct knowledge than patients with low to medium education (31.9% versus 13.9%, P < 0.001). However, stroke awareness was not different by educational status (33.6% versus 36.2%, P = 0.609).

**Table 1:** Baseline characteristics of patients

Characteristics	
Age, years	67 (57-73)*
Gender, male (%)	308 (62)
Time to hospital arrival, minutes	474 (170-1313)*
NIHSS, score	3 (2-6)*
Risk factors	
Previous stroke (%)	116 (23)
Hypertension (%)	316 (63)
Diabetes (%)	134 (27)
Hyperlipidemia (%)	72 (14)
Current smoker (%)	150 (30)
Atrial fibrillation (%)	44 (9)
Coronary heart disease (%)	38 (8)
Family history of stroke (%)	147 (29)
Study hospital located in Seoul (%)	324 (65)
Education	
Low (0-6 years) (%)	169 (34)
Medium (6-12 years) (%)	212 (42)
High (≥12 years) (%)	116 (23)
Living alone (%)	66 (13)
Visit hospital regularly (%)	338 (68)
Presence of bystander at time of symptom onset (%)	355 (71)
Arrival through referral (%)	169 (34)
Mode of transport	
Ambulance (%)	182 (36)
Personal vehicle (%)	200 (40)
Public transportation (%)	113 (23)
Knowledge by patient of thrombolysis (%)	92 (18)
Awareness of the patient/bystander that the initial symptom was stroke related (%)	179 (36)

N = 500

*Median (25^th^-75^th ^percentile)

Table 2 presents prehospital delay time by explanatory variables such as NIHSS score, risk factors and general patient characteristics. Shorter times were significantly associated with high NIHSS scores, atrial fibrillation, use of an ambulance, and awareness by patient/bystander that the initial symptom was stroke-related. Diabetes, hyperlipidemia and referral from other hospitals were associated with longer times.

**Table 2:** Factors associated with increased time from symptom onset to hospital arrival: univariate analysis

Variables	N (%)	Prehospital delay	P
Clinical			
NIHSS, score			< 0.001
0-3	261 (52)	595 (242-1452)	
4-6	136 (27)	573 (244-1357)	
≥ 7	103 (21)	170 (55-508)	
Pre-morbid disability (mRS > 2)			0.798
Yes	18 (4)	463 (126-1196)	
No	482 (96)	471 (170-1308)	
Risk factors			
Previous stroke			0.088
Yes	116 (23)	316 (110-1241)	
No	384 (77)	502 (210-1338)	
Hypertension			0.632
Yes	316 (63)	540 (165-1305)	
No	184 (37)	405 (170-1317)	
Diabetes			0.049
Yes	134 (27)	568 (234-1460)	
No	366 (73)	449 (148-1253)	
Hyperlipidemia			0.039
Yes	72 (14)	753 (236-1391)	
No	428 (76)	432 (152-1297)	
Current smoker			0.637
Yes	150 (30)	466 (196-1227)	
No	350 (70)	504 (150-1344)	
Atrial fibrillation			0.002
Yes	44 (9)	215 (59-948)	
No	456 (91)	521 (194-1328)	
Coronary heart disease			0.067
Yes	38 (8)	277 (69-1012)	
No	462 (92)	504 (180-1314)	
Family history of stroke			0.171
Yes	147 (29)	762 (327-1409)	
No	353 (71)	682 (230-1353)	
General			
Location of study hospital			0.096
Seoul	324 (65)	558 (165-1365)	
Metropolitan area	176 (35)	397 (170-1126)	
Education			0.572
Low (0-6 years)	169 (34)	444 (170-1294)	
Medium (6-12 years)	215 (43)	551 (210-1308)	
High (≥ 12 years)	116 (23)	379 (122-1307)	
Living alone			0.322
Yes	435 (87)	506 (283-1348)	
No	65 (13)	462 (150-1309)	
Visit hospital regularly			0.503
Yes	338 (68)	504 (152-1248)	
No	162 (32)	466 (183-1350)	
Presence of bystander at time of symptom onset			0.742
Yes	145 (29)	502 (170-1358)	
No	355 (71)	470 (170-1252)	
Arrival through referral			< 0.001
Yes	169 (34)	847 (355-1575)	
No	331 (66)	347 (106-1015)	
Mode of transport			< 0.001
Ambulance	182 (36)	301 (79-758)	
Personal vehicle	205 (41)	608 (242-1373)	
Public transportation	113 (23)	858 (290-1620)	
Knowledge by patient of thrombolysis			0.132
Yes	92 (18)	349 (113-1121)	
No	408 (82)	502 (208-1328)	
Awareness of the patient/bystander that the initial symptom was stroke related			< 0.001
Yes	179 (36)	260 (100-708)	
No	321 (64)	650 (290-1458)	

Values are median (25^th^-75^th ^percentile) and are given in minutes.

*Mann-Whitney U test or Kruskal-Wallis test was used.

Analysis of variables according to arrival time (Table 3) showed that previous stroke history and knowledge about thrombolysis were also related to early arrival. However, diabetes and hyperlipidemia were no longer significantly associated with late arrival.

**Table 3:** Distribution of clinical characteristics according to arrival time

Characteristics	Early arrival (≤ 3 hours) (n = 132)	Late arrival (> 3 hours) (n = 368)	P
Age, years	68 (57-73)^†^	66 (57-74)^†^	1.000*
Gender, male (%)	80 (61)	228 (62)	0.784
NIHSS, score	5 (2-10)^†^	3 (1-5)^†^	< 0.001*
Pre-morbid disability (mRS >2) (%)	7 (5)	11 (3)	0.232
Risk factors			
Previous stroke (%)	43 (33)	73 (20)	0.003
Hypertension (%)	85 (64)	231 (63)	0.740
Diabetes (%)	27 (21)	107 (29)	0.055
Hyperlipidemia (%)	13 (10)	59 (16)	0.083
Current smoker (%)	35 (27)	115 (31)	0.308
Atrial fibrillation (%)	21 (16)	23 (6)	0.001
Coronary heart disease (%)	15 (11)	23 (6)	0.057
Family history of stroke (%)	35 (27)	112 (30)	0.396
Study hospital located in Seoul (%)	84 (64)	240 (65)	0.744
Highly educated (≥12 years) (%)	36 (27)	80 (22)	0.196
Living alone (%)	11 (8)	55 (15)	0.054
Visit hospital regularly (%)	91 (69)	247 (67)	0.702
Presence of bystander at time of symptom onset (%)	92 (70)	263 (72)	0.701
Arrival through referral (%)	15 (11)	154 (42)	< 0.001
Arrival by Ambulance (%)	71 (54)	111 (30)	< 0.001
Knowledge by patient of thrombolysis (%)	34 (26)	58 (16)	0.011
Awareness of the patient/bystander that the initial symptom was stroke related (%)	75 (57)	104 (28)	< 0.001

Pearson's χ^2 ^test and the *Mann-Whitney *U *test were used

^†^Median (25^th^-75^th ^percentile)

Multivariable logistic regression analysis (Table 4) identified the variables that were independently associated with early arrival. Among them, awareness by the patient/bystander that the initial symptom was stroke-related was highly associated with early arrival (OR, 4.438; 95% CI, 2.669-7.381). Knowledge about thrombolysis (OR, 2.002; 95% CI, 1.104-3.633), use of ambulance (OR, 1.961; 95% CI, 1.176-3.270) and high NIHSS score (OR, 1.101; 95% CI, 1.038-1.167) were also independently associated with early arrival, and referral from another hospital (OR, 0.116; CI, 0.059-0.228) was independently associated with late arrival.

**Table 4:** Multivariable logistic regression analysis: factors independently associated with early arrival

	Arrival time (≤ 3 hours)		
		
	Crude OR (95% CI)	Adjusted OR (95% CI)	P*
NIHSS	1.152 (1.099-1.207)	1.101 (1.038-1.167)	0.001
Previous stroke	1.952 (1.251-3.047)	1.185 (0.680-2.067)	0.549
Diabetes	0.627 (0.388-1.013)	0.642 (0.367-1.122)	0.120
Hyperlipidemia	0.572 (0.303-1.081)	0.485 (0.227-1.035)	0.061
Atrial fibrillation	2.838 (1.513-5.323)	2.152 (0.975-4.747)	0.058
Coronary heart disease	1.923 (0.971-3.809)	0.882 (0.362-2.146)	0.781
Living alone	1.933 (0.979-3.817)	1.710 (0.767-3.810)	0.190
Arrival through referral	0.178 (0.100-0.317)	0.116 (0.059-0.228)	< 0.001
Arrival by Ambulance	2.697 (1.790-4.063)	1.961 (1.176-3.270)	0.010
Knowledge by patient of thrombolysis	1.854 (1.147-2.998)	2.002 (1.104-3.633)	0.022
Awareness of the patient/bystander that the initial symptom was stroke related	3.340 (2.211-5.045)	4.438 (2.669-7.381)	< 0.001

*P for multivariate model

Candidate variables were selected from the results of univariate analysis with P < 0.2

## Discussion

Despite the results of previous studies that showed that early arrival is not associated with patients' knowledge about stroke [8-10], it is still believed that awareness of stroke as a severe symptom may lead to shorter delay times and increased thrombolysis rates [11,12]. In our study, stroke awareness and knowledge about thrombolysis were independently associated with lower prehospital delay. Interestingly, awareness of the patient/bystander that the initial symptom was stroke-related was the factor most strongly associated with early arrival. This result may suggest that an immediate response not only by the patient, but also by the bystander, is important for early arrival. However, only 178 patients (36%), even including bystanders' knowledge, knew that the patient had had a stroke, and only 92 patients (18%) had knowledge of thrombolysis. Even though these results may appear disappointing, the opportunity exists to increase public knowledge and thrombolysis rates. Furthermore, the finding that only one-third of patients had heard of thrombolysis from their doctors was surprising, and doctors in Korea should make greater efforts to educate patients.

Our study confirmed results from previous reports that educational level was not associated with early arrival [13,14]. However, we could speculate that the bystander's educational level may potentially affect early arrival. In our data, highly educated patients were more informed about thrombolysis, and patients who had knowledge about thrombolysis presented to the ED earlier. Nevertheless, patients' educational level was not associated with early arrival. It may be that statistical significance was lost because the patients who were accompanied by highly educated bystanders arrived faster because of the bystanders' knowledge of stroke symptoms. Therefore, we suggest that the bystander's knowledge and behavior after stroke onset may greatly impact early arrival. Because there are many elderly people in Korea with little education who live together with their descendants, the knowledge and behavior of bystanders may be more important than those of patients.

Similar to many other studies [8,9,15-18], we found that severe stroke and use of ambulance were significantly associated with early arrival, and referral from other hospitals was negatively associated with it. We also found that patients who were referred from other hospitals never used EMS when they visited primary care centers. Interestingly, 37 patients who were referred by primary Oriental physicians had longer prehospital delay times (median, 1214 minutes) than patients who came from Western physicians (median, 730 minutes). Considering that patients who arrived after 48 hours were not included in this study, the delay time of patients who were referred by primary Oriental physicians may actually be greater. In Korea, traditional Oriental medicine is very familiar to the general public, especially to the elderly and subjects with little education [7]. In order to increase the thrombolysis rate and better stroke outcome, publicity focusing on these subjects is needed.

Demographic and clinical factors such as age, sex, pre-morbid disability, risk factors, living alone, visiting the hospital regularly, and presence of a bystander, were not associated with early arrival in multivariate analyses, consistent with other reports [19-21]. In one study, recognition of symptoms by a witness was related to early arrival [21], but in our data, presence of a bystander on its own did not have any significance. These findings may indicate that the presence of witnesses as well as their knowledge and behavior are important. Care sought after stroke symptom onset was associated with the previous stroke history of patients in one study [22], but it did not remain significant after multivariate analysis, suggesting that more education of patients during hospitalization is required.

Although this study was conducted prospectively in 14 tertiary hospitals, it has a few limitations. First, although Seoul and its metropolitan area include almost half the population of Korea, it includes few rural regions. In fact, the metropolitan area in this study consists of more than 1 million people, and their socio-economic status is not very different from that of the patients in Seoul. Therefore, there might be some economic, educational and socio-psychological factor biases. Second, we excluded patients who could not be interviewed (severe symptoms, death), and those who received thrombolysis therapy outside the hospital. These exclusion criteria may lead to slightly increased delay times and decreased median NIHSS scores, because patients with severe symptoms tend to arrive earlier. In addition, we only included patients who arrived within 48 hours from symptom onset, and this may also affect the overall delay time. However, we only investigated patients who arrived within 48 hours because they are more likely to be candidates for thrombolysis.

## Conclusion

This is the first multicenter study to investigate factors associated with prehospital delay in Korea and to find that awareness of stroke symptoms on the part of patient and bystander is the most important factor in early arrival. Additionally, we found that knowledge about thrombolysis and transportation by ambulance may shorten prehospital delay. Therefore, widespread and repeated public education via the media is needed to improve recognition of stroke symptoms and ensure the appropriate response. Furthermore, baseline surveys of public awareness of stroke should also be carried out to evaluate current knowledge.

## Competing interests

The authors declare that they have no competing interests.

## Authors' contributions

YSK interpreted the data and drafted the manuscript. SSP, KK, SL and SHL participated in the design of the study and intellectual discussion of the results. HJB, AHC, YJC, MKH, JHH, DEK, HYK, GMK, SUK, HMK, BCL, KBL, YSL, HSN, MSO, JMP, JHR and KHY are principle investigators of each hospital. They participated in the study design and competitively recruited patients. BWY conceived the study, and participated in its design and coordination. He recruited patients, contributed to the intellectual discussion of the concept of the article and gave final approval of the version to be published. All authors read and approved the final manuscript. Each author has participated sufficiently in the work to take public responsibility for appropriate portions of the content.

## Pre-publication history

The pre-publication history for this paper can be accessed here:

http://www.biomedcentral.com/1471-2377/11/2/prepub
